# Stochastic convex sparse principal component analysis

**DOI:** 10.1186/s13637-016-0045-x

**Published:** 2016-09-09

**Authors:** Inci M. Baytas, Kaixiang Lin, Fei Wang, Anil K. Jain, Jiayu Zhou

**Affiliations:** 1 Computer Science and Engineering, Michigan State University, East Lansing, 48824 USA; 2 School of Software Engineering, Beijing University of Technology, Beijing, China

**Keywords:** Sparse PCA, Convex PCA, Proximal mapping

## Abstract

Principal component analysis (PCA) is a dimensionality reduction and data analysis tool commonly used in many areas. The main idea of PCA is to represent high-dimensional data with a few representative components that capture most of the variance present in the data. However, there is an obvious disadvantage of traditional PCA when it is applied to analyze data where interpretability is important. In applications, where the features have some physical meanings, we lose the ability to interpret the principal components extracted by conventional PCA because each principal component is a linear combination of all the original features. For this reason, sparse PCA has been proposed to improve the interpretability of traditional PCA by introducing sparsity to the loading vectors of principal components. The sparse PCA can be formulated as an *ℓ*_1_ regularized optimization problem, which can be solved by proximal gradient methods. However, these methods do not scale well because computation of the exact gradient is generally required at each iteration. Stochastic gradient framework addresses this challenge by computing an expected gradient at each iteration. Nevertheless, stochastic approaches typically have low convergence rates due to the high variance. In this paper, we propose a convex sparse principal component analysis (Cvx-SPCA), which leverages a proximal variance reduced stochastic scheme to achieve a geometric convergence rate. We further show that the convergence analysis can be significantly simplified by using a weak condition which allows a broader class of objectives to be applied. The efficiency and effectiveness of the proposed method are demonstrated on a large-scale electronic medical record cohort.

## Introduction

Principal component analysis (PCA) is a commonly used dimensionality reduction and data analysis tool in many areas such as computer vision [1, 2], data mining [3, 4], biomedical informatics [5, 6], and many others. The goal of PCA is to learn a linear transformation such that the learned principal components are the dimensions retaining the most of the variance in the data. Principal components are obtained by computing the eigenvalue decomposition of the covariance matrix, and it can also be computed by the singular value decomposition of the data matrix. Let $\mathbf {S} = \frac {1}{n} \sum _{i = 1}^{n} x_{i}{x_{i}^{T}}$ be the normalized covariance matrix for *n* training data points where each data point is in a *d*-dimensional feature space. The PCA of computing the top *p* components can be written as the following optimization problem:

1 $$\begin{array}{*{20}l} \max_{\mathbf{Z} \in \mathbb{R}^{d \times p}} \left\|\mathbf{SZ}\right\|_{F}^{2}, \quad \text{s.t.} \mathbf{Z}^{T}\mathbf{Z} = \mathbf{I}, \end{array} $$

where **Z** is an orthogonal projection matrix. In many applications, we are only interested in a few top principal components. In this case, the principal components can be computed in an iterative fashion: the leading principal component is calculated at each iteration (e.g., using power methods), and we then deflate the computed component and the next principal component now becomes the leading one [7]. Therefore, we focus on finding the leading principal component in this paper. In spite of its advantages, there is an obvious disadvantage of PCA. In the solution of Eq. (1), the principal components are linear combinations of all input variables. This means that the columns of **Z** matrix, which are called loadings of principal components, are dense. One important implication of dense loadings is that we lose the ability to interpret the output dimensions of conventional PCA. PCA works well if we are not interested in the physical meanings of the features or if the interpretation of principal components is not crucial for the application. However, the intepretability is a significant factor when it comes to many applications such as biology, finance, and biomedical informatics. In the domain of biomedical informatics, as more and more electronic medical records (EMR) [8] of patients are available, medical researchers are interested in applying various techniques to analyze the EMR data. Each feature of the EMR data is a record/event related to a certain diagnosis. When the traditional PCA is applied to the data, those medical features are projected to a low dimensional space, in which each new feature will be the linear combination of all the original features. In this case, it is hard to comprehend the meaning of the new features.

Sparse PCA has been proposed to address this drawback. In sparse PCA, we learn sparse loading vectors which combine only few of the input variables allowing interpretation of the principal components. Sparse PCA was firstly proposed by Zou et al. in [9], where PCA was formulated as a regression problem and the sparse PCA was introduced by imposing the lasso (elastic net) constraint. Other common approaches to solve the sparse PCA problem are semi-definite programming [10, 11] and inverse power method [12]. Moreover, a more recent study [13] investigated sparse PCA with oracle property. Aforementioned approaches are generally not scalable enough to work with large-scale datasets. One way to deal with large sample sizes is using stochastic methods. We can see an example of stochastic PCA in [7]. Authors described an algorithm with computationally cheap stochastic iterations and variance reduction which was suggested in [14].

In this study, sparse PCA is posed as an *ℓ*_1_ regularized optimization problem. Standard approaches to solve such sparse learning problems are proximal gradient methods [15–17], which require computation of the full gradient at each iteration. These methods generally work with a composite function including a smooth part and a non-smooth part. A large family of machine learning problems [18–23] can be expressed as composite functions. Traditionally, solving problems with objectives, which are not continuously differentiable, requires subgradient descent [24] which has very poor performance [25]. The recently developed proximal gradient methods can solve these composite problems with fast convergence rates [26, 27]. However, these methods are hardly scalable to large-scale problems with large sample sizes because of the computation of full gradient. Therefore, stochastic gradient-based methods are preferred in such problems. One major disadvantage of the stochastic gradient descent is the low convergence due to high variance by random sampling. Johnson and Zhang proposed a solution for this drawback in [14]. Their solution reduced the variance by using a copy of the estimated optimal point and the full gradient at this point in the gradient step. This approach exploited the strong convexity property to obtain a geometric convergence rate under expectation. Xiao and Zhang similarly presented a multi-stage scheme to progressively reduce the variance of the proximal stochastic gradient (Prox-SVRG) with a geometric convergence rate under expectation in [15]. The fundamental assumptions were Lipschitz continuity of the gradient of smooth part and the strong convexity of the objective function.

To tackle the aforementioned challenges in this paper, we introduce a novel stochastic convex sparse PCA (Cvx-SPCA) method which is extremely efficient and can handle large-scale datasets. Specifically, we propose to adopt a convex formulation of PCA [28] which provides a strongly convex function. The problem structure in this design allows us to leverage efficient scheme of Prox-SVRG [15] which leads to an exponential (geometric) convergence rate. We also investigate the convergence analysis of Prox-SVRG and present a new proof of the convergence rate which significantly reduces the conditions and assumptions required. As such, we show that the optimization scheme can be applied to a much larger class of problems to obtain the geometric convergence rate. We conducted extensive experiments on both synthetic and real datasets to illustrate the efficiency of the proposed algorithm. Because of its efficiency, we were able to apply the proposed algorithm to analyze a real EMR cohort with a large number of patients, which is hardly possible to analyze by using traditional approaches.

## Convex sparse principal component analysis

In this section, we introduce the problem formulation and optimization scheme of the proposed approach. The problem of finding a sparse loading vector is posed as the combination of *ℓ*_1_ sparsity inducing norm and convexity from the convex principal component analysis, which allows us to utilize an extremely efficient stochastic proximal gradient approach.

### Convex sparse PCA

The goal of sparse PCA is to learn sparse loading vectors such that the principal components will be linear combinations of a few key variables instead of all the variables. We propose the following convex optimization problem:

2 $$\begin{array}{*{20}l} \min_{\mathbf{z} \in \mathbb{R}^{d}} \left\{P\left(\mathbf{z}\right) = F\left(\mathbf{z}\right) + R\left(\mathbf{z}\right)\right\},  \end{array} $$

where the convex PCA loss [28] is given by:

$$F\left(\mathbf{z}\right) = \tfrac{1}{2} \mathbf{z}^{T}\left(\lambda \mathbf{I} - \mathbf{S}\right)\mathbf{z} -\mathbf{w}^{T}\mathbf{z} $$

and the regularization term *R*(**z**)=*γ*∥**z**∥_1_ is the *ℓ*_1_ norm of the loading vector **z**, $\gamma \in \mathbb {R}$ is the regularization parameter controlling the sparsity of the loading vector, *λ*>*λ*_1_(**S**) is the convexity parameter, $\mathbf {w} \in \mathbb {R}^{d}$ is a random vector, and $\mathbf {S} = \frac {1}{n} \sum _{i = 1}^{n} \mathbf {x}_{i}\mathbf {x}_{i}^{T}$. Here, *λ*_1_(**S**) represents the largest eigenvalue of the covariance matrix **S** and **w** is a vector of normally distributed random numbers. An upper bound for the regularization term *γ* can be derived by using standard subgradient analysis [25]: if the regularization parameter *γ* is larger than the maximum of absolute value of the elements of the vector **w**, i.e., ∥**w**∥_*∞*_, we will end up with trivial solutions (solutions with only zeros). This thus guides us to use a parameter range of *γ*∈[0,∥**w**∥_*∞*_].

In the above approach, we use a convex optimization formulation of finding the first principal component inspired by the work in [28]. Even though *R*(**z**) is not strongly convex, the overall cost function in Eq. (2) is a strongly convex function in which the strong convexity comes from *F*(**z**). The structure of the problem defined in Eq. (2) allows us to use gradient based algorithms to obtain the global solution. Moreover, the strong convexity usually ensures nice convergence properties for stochastic gradient schemes as well. Therefore, we can also benefit from the faster convergence rate of the proximal stochastic scheme proposed in [15]. We note that the objective function of traditional PCA as shown in Eq. (1) does not define a convex problem, and thus, the analysis in this paper cannot be applied to it.

The most common methods to solve problems such as Eq. (2), where the objective function is comprised of the average of smooth component functions and a non-smooth function, are proximal gradient methods. In the next section, the method used to solve convex optimization problem given in Eq. (2) will be explained.

### Optimization scheme

In this paper, we propose to use a proximal stochastic gradient method with progressive variance reduction approach [15] to solve the problem in Eq. (2). The function denoted by *F*(**z**) can also be written as the sum of *n* smooth functions:

3 $$ F\left(\mathbf{z}\right) = \frac{1}{n}{\sum\nolimits}_{i = 1}^{n} \frac{1}{2} \mathbf{z}^{T} \left(\lambda \mathbf{I} - \mathbf{x}_{i}\mathbf{x}_{i}^{T}\right) \mathbf{z} -\mathbf{w}^{T}\mathbf{z}.   $$

When *n* is very large, calculating the full gradient at each gradient descent iteration is an expensive operation. Hence, stochastic gradient methods are preferred to solve such problems. In stochastic approach, instead of calculating gradients for all of the data points, one data point is randomly sampled and the gradient at this point is calculated at each iteration. Therefore, the number of calculations decreases. However, the drawback of the stochastic gradient methods is the high variance introduced because of random sampling. As a result of the high variance, we suffer from poor convergence rates. As discussed previously, there are solutions to reduce the variance and increase the convergence rate. One of the studies which mitigates the high variance problem of stochastic gradient method is proximal stochastic gradient method with progressive variance reduction [15]. The study in [15] showed that the variance of the gradient can be upper bounded by using a multi-stage scheme which progressively reduces the variance. When the algorithm converges to optimal point, variance also converges to zero. Therefore, this approach can achieve better convergence rates than conventional stochastic gradient even with constant step sizes. We refer the readers to Section 3.1 in [15] for detailed proof of bounding the variance.

In this paper, we also follow the approach in [15]. The algorithm used in this study is given in Algorithm 1.



In the algorithm, **z**_0_ is the initial value for loading vector **z**, *η* is the constant step size, *γ* is the regularization term to control sparsity of **z**, *m* is the number of iterations for each epoch *s*, and *T* is the maximum number of epochs. At each epoch, full gradient at the point $\tilde {\mathbf {z}}$ is calculated periodically. The cost of calculating the full gradient is the product of a *d*×*d* matrix and a *d* dimensional vector. Therefore, the most time consuming part in our algorithm is the multiplications with covariance matrix, when the feature dimension is high. $\tilde {\mathbf {z}}$ is an estimate of the optimal point and it is updated at each epoch to be utilized in gradient calculations. During *m* stochastic gradient steps, we first sample a data point randomly and compute the gradient **v**_*k*_. If we take the expectation of the gradient calculated in Eq. (4), we can see that **v**_*k*_ is also an estimate of the full gradient as in conventional stochastic gradient methods. This shows that **v**_*k*_ given below is in the same direction as the full gradient under expectation.

4 $$ \begin{aligned} \mathbf{v}_{k} &= \nabla f_{ik}\left(\mathbf{z}_{k-1}\right) - \nabla f_{ik}\left(\tilde{\mathbf{z}}\right) + \nabla F\left(\tilde{\mathbf{z}}\right) \\ &= \left(\lambda \mathbf{I} - x_{ik}x_{ik}^{T}\right)\left(\mathbf{z}_{k-1} - \tilde{\mathbf{z}}\right) + \left(\lambda \mathbf{I} - \mathbf{S}\right)\tilde{\mathbf{z}} - \mathbf{w}, \end{aligned}   $$

where $\nabla F\left (\tilde {\mathbf {z}}\right)$ is the average gradient of functions *f*_*i*_(**z**),*i*=1,…,*n* or the full gradient at point $\tilde {\mathbf {z}}$, ∇*f*_*ik*_(**z**_*k*−1_) is the gradient of the function calculated by using the data point *x*_*ik*_ sampled at the *k*th iteration and $\tilde {\mathbf {z}}$ is the average of *z*_*k*_, *k*=1,..,*m* at the end of an epoch.

After the gradient computation, we update **z**_*k*_ by using the proximal mapping for *ℓ*_1_ norm as follows.

$$\begin{array}{*{20}l} \mathbf{z}_{k} &= \text{prox}_{\eta,\gamma}\left(\mathbf{z}_{k-1} - \eta \mathbf{v}_{k}\right) \\ &= \text{sign}\left(\mathbf{z}_{k-1} - \eta \mathbf{v}_{k}\right) \max\left(0,|\mathbf{z}_{k-1} - \eta \mathbf{v}_{k}|-\eta \gamma\right). \end{array} $$

In this algorithm, variance of the stochastic gradient **v**_*k*_ is reduced progressively, while both $\tilde {\mathbf {z}}$ and **z**_*k*−1_ are converging to the optimal point *z*_∗_= arg min**zP**(**z**) [15]. Since the full gradient is utilized to modify stochastic gradients and function *F* is an average of smooth component functions, variance can be bounded. In the next section, we will give the convergence analysis of the aforementioned algorithm.

## Convergence analysis

In this section, we present the convergence analysis of the proposed algorithm. The objective function used in this paper is suitable to follow the convergence analysis in [15]. Therefore, our analysis is mostly adapted from [15]. However, we use much weaker conditions which allow a broader family of objective functions to fit in this scheme and to enjoy the geometric convergence. We retain the following assumption used throughout in [15]:

### **Assumption 1**

The function *R*(**z**) is lower semi-continuous and convex, and its effective domain, $dom(R):=\left \{\mathbf {z}\in \mathbb {R}^{d} | R\left (\mathbf {z}\right)<+\infty \right \}$ is closed. Each *f*_*i*_(**z**), *for* *i*=1,…,*n*, is differentiable on an open set that contains *dom*(*R*), and their gradients are Lipschitz continuous. That is, there exist *L*_*i*_>0 such that for all **z**,**y**∈*dom*(*R*),

$$ \left\|\nabla f_{i}\left(\mathbf{z}\right) - \nabla f_{i}\left(\mathbf{y}\right)\right\| \leq L_{i}\left\|\mathbf{z}-\mathbf{y}\right\|, $$

which also implies that the gradient of the average function *F*(**z**) is also Lipschitz continuous, i.e., there is an *L*>0 such that for all **z**,**y**∈*dom*(*R*),

$$\left\|\nabla F\left(\mathbf{z}\right) - \nabla F\left(\mathbf{y}\right) \right\|\leq L\left\|\mathbf{z}-\mathbf{y}\right\|, $$

where $L \leq \left (1/n\right)\sum _{i = 1}^{n}L_{i}$.

In [15], convergence analysis was done for general *F* and *R* functions and both of them were assumed to be strongly convex. On the other hand, we only assume that functions *F*(**z**) and *R*(**z**) are convex, but not necessarily strongly convex. Thus, we are relaxing this strong assumption at this point. Strong convexity provides good properties and is relevant for faster convergence rates. However, objective functions are not always strongly convex in many cases. Therefore, a simplified version of the analysis will be preferable, when the objective functions do not have necessarily strong convexity property.

Although our overall objective function is strongly convex, *R*(**z**) is not strongly convex as it was mentioned in the previous section. Therefore, we drop the strong convexity assumption at two steps in the original analysis of [15] and obtain the convergence rate given in the following theorem.

### **Theorem 1**

Under the assumption that Assumption 1 holds and 0<*η*<1/(4*L*_*Q*_), where *L*_*Q*_=max_*i*_ *L*_*i*_, the convergence rate is obtained as follows:

5 $$ \begin{aligned} & \rho = \frac{1}{\ell \left(1-4L_{Q}\eta\right)m\eta} + \frac{4L_{Q}\eta \left(m+1\right)}{\left(1-4L_{Q}\eta\right)m} < 1, \\ & \mathbb{E}\left\{P\left(\tilde{\mathbf{z}}_{s}\right)\right\} - P\left(\mathbf{z}_{*}\right) \leq \rho^{s} \left[P\left(\tilde{\mathbf{z}}_{0}\right) - P\left(\mathbf{z}_{*}\right)\right], \end{aligned}   $$

where *z*_∗_= arg min**zP**(**z**).

### *Proof*

The proof of Theorem 1 starts with investigating the distance between *z*_*k*_ and *z*_∗_; ∥*z*_*k*_−*z*_∗_∥^2^. According to the stochastic gradient mapping definition in [15], *z*_*k*_ can be written as *z*_*k*−1_−*η* *g*_*k*_.

6 $$ \begin{aligned} \left\|\mathbf{z_{k}} - \mathbf{z_{*}}\right\|^{2} &= \left\|\mathbf{z_{k-1}} - \eta \mathbf{g_{k}} -\mathbf{z_{*}}\right\|^{2} \\ &= \left\|\mathbf{z_{k-1}} - \mathbf{z_{*}}\right\|^{2} - 2\eta \mathbf{g_{k}}^{T}\left(\mathbf{z_{k-1}-\mathbf{z_{*}}}\right) \\ &\quad+ \eta^{2} \left\| \mathbf{g_{k}}\right\|^{2}. \end{aligned}  $$

The term $\left (- \mathbf {g_{k}}^{T}\left (\mathbf {z_{k-1}-\mathbf {z_{*}}}\right) + \frac {\eta }{2} \left \| \mathbf {g_{k}}\right \|^{2}\right)$ can be bounded by using the definition of the proximal update as shown below.

$$\begin{array}{*{20}l} \mathbf{z_{k}} &= \text{prox}_{\eta R} \left(\mathbf{z_{k-1}} - \eta \mathbf{v_{k}}\right) \\ & = \arg\min_{y} \left\{\frac{1}{2} \left\|\mathbf{y} - \left(\mathbf{z_{k-1}}-\eta \mathbf{v_{k}}\right) \right\|^{2} + \eta R\left(\mathbf{y}\right)\right\} \end{array} $$

According to the optimality condition,

$$\begin{array}{*{20}l} \mathbf{z_{k}} - \left(\mathbf{z_{k-1}} - \eta \mathbf{v_{k}}\right) + \eta \xi = 0, \end{array} $$

where *ξ*∈*∂* *R*(*z*_*k*_) is the subgradient of *R*(**z**) at *z*_*k*_. If we combine the stochastic gradient mapping definition with the optimality condition, we obtain the following expression.

$$\begin{array}{*{20}l} \mathbf{z_{k}} - \left(\mathbf{z_{k}} + \eta \mathbf{g_{k}} - \eta \mathbf{v_{k}}\right) + \eta \xi = 0 \Rightarrow \xi = \mathbf{g_{k}} - \mathbf{v_{k}} \end{array} $$

By using the convexity of *F*(**z**) and *R*(**z**), we can write the following inequality.

7 $$  \begin{aligned} P\left(\mathbf{y}\right) &= F\left(\mathbf{y}\right) + R\left(\mathbf{y}\right) \\ &\geq F\left(\mathbf{z_{k-1}}\right) + \nabla F\left(\mathbf{z_{k-1}}\right)^{T} \left(\mathbf{y} - \mathbf{z_{k-1}}\right) \\ &\quad+ R\left(\mathbf{z_{k}}\right) + \xi^{T} \left(\mathbf{y} - \mathbf{z_{k}}\right) \end{aligned}  $$

Convergence analysis of [15] utilized strong convexity of *F* and *R* in 7. However, we will show that strong convexity is not required at this point. Since *F*(**z**) is assumed to be Lipschitz continuous with Lipschitz constant *L*, *F*(*z*_*k*−1_) can also be bounded by using Theorem 2.1.5 in [29].

8 $$  \begin{aligned} F\left(\mathbf{z_{k-1}}\right) &\geq F\left(\mathbf{z_{k}}\right) - \nabla F\left(\mathbf{z_{k-1}}\right)^{T} \left(\mathbf{z_{k}} - \mathbf{z_{k-1}}\right) \\ &\quad- \frac{L}{2} \left\|\mathbf{z_{k}}-\mathbf{z_{k-1}}\right\|^{2} \end{aligned}  $$

If we combine Eqs. (7) and (8), we obtain the following inequality.

$$\begin{array}{*{20}l} P\left(\mathbf{y}\right) &\geq F\left(\mathbf{z_{k}}\right) - \nabla F\left(\mathbf{z_{k-1}}\right)^{T} \left(\mathbf{z_{k}} - \mathbf{z_{k-1}}\right) \\ &\quad- \frac{L}{2}\left\|\mathbf{z_{k}}-\mathbf{z_{k-1}}\right\|^{2} + \nabla F\left(\mathbf{z_{k-1}}\right)^{T} \left(\mathbf{y}-\mathbf{z_{k-1}}\right) \\ &\quad+ R\left(\mathbf{z_{k}}\right) + \xi^{T} \left(\mathbf{y} - \mathbf{z_{k}}\right) \\ & \geq P\left(\mathbf{z_{k}}\right) - \nabla F\left(\mathbf{z_{k-1}}\right)^{T}\left(\mathbf{z_{k}} - \mathbf{z_{k-1}}\right) \\ &\quad- \frac{L}{2}\left\| \mathbf{z_{k}} - \mathbf{z_{k-1}} \right\|^{2} +\nabla F\left(\mathbf{z_{k-1}}\right)^{T}\left(\mathbf{y} - \mathbf{z_{k-1}}\right) \\ &\quad+ \xi^{T} \left(\mathbf{y} - \mathbf{z_{k}}\right) \end{array} $$

Here, we again use stochastic gradient mapping; *z*_*k*_−*z*_*k*−1_=−*η* *g*_*k*_ to obtain the following inequality.

$$\begin{array}{*{20}l} P\left(\mathbf{y}\right) &\geq P\left(\mathbf{z_{k}}\right) + \nabla F\left(\mathbf{z_{k-1}}\right)^{T} \left(\mathbf{y} - \mathbf{z_{k}}\right) \\ & \quad+ \xi^{T} \left(\mathbf{y} - \mathbf{z_{k}}\right) - \frac{L}{2}\eta^{2} \left\|\mathbf{g_{k}}\right\|^{2} \end{array} $$

If we substitute *ξ* with *g*_*k*_−*v*_*k*_, then add and subtract *z*_*k*−1_ from the term (**y**−*z*_*k*_):

$$\begin{array}{*{20}l} P\left(\mathbf{y}\right) &\geq P\left(\mathbf{z_{k}}\right) + \left(\mathbf{v_{k}} - \nabla F\left(\mathbf{z_{k-1}}\right)\right)^{T} \left(\mathbf{z_{k}} - \mathbf{y}\right) \\ &\quad+ \mathbf{g_{k}}^{T} \left(\mathbf{y} + \mathbf{z_{k-1}} - \mathbf{z_{k-1}} - \mathbf{z_{k}}\right) -\frac{L}{2}\eta^{2} \left\|\mathbf{g_{k}}\right\|^{2} \end{array} $$

$$\begin{array}{*{20}l} P\left(\mathbf{y}\right) &\geq P\left(\mathbf{z_{k}}\right) + \mathbf{g_{k}}^{T} \left(\mathbf{y} - \mathbf{z_{k-1}}\right) + \left(\eta - \frac{L}{2}\eta^{2}\right)\left\|\mathbf{g_{k}}\right\|^{2} \\ &\quad+\left(\mathbf{v_{k}} - \nabla F\left(\mathbf{z_{k-1}}\right)\right)^{T} \left(\mathbf{z_{k}} - \mathbf{y}\right) \end{array} $$

Under the assumption of 0<*η*<1/4*L*_*Q*_<1/*L*, $\left (\eta - \frac {L}{2}\eta ^{2}\right) = \frac {\eta }{2}\left (2 - L\eta \right)$ can be taken as *η*/2. Because (2−*L* *η*) is between (1,2) according to the assumption, therefore, eliminating (2−*L* *η*) does not change the inequality. Now we will use the result derived above for the term $\left (-\mathbf {g_{k}}^{T} \left (\mathbf {z_{k-1} - z_{*}}\right) + \frac {\eta }{2}\left \|\mathbf {g_{k}}\right \|^{2}\right)$ in Eq. (6).

9 $$ \begin{aligned} \left\|\mathbf{z_{k}} - \mathbf{z_{*}}\right\|^{2} &\leq \left\|\mathbf{z_{k-1}} - \mathbf{z_{*}}\right\|^{2} + 2\eta \left(P\left(\mathbf{z_{*}}\right) - P\left(\mathbf{z_{k}}\right)\right) \\ &\quad- 2\eta \Delta^{T}\left(\mathbf{z_{k}}-\mathbf{z_{*}}\right), \end{aligned}  $$

where *Δ*=*v*_*k*_−∇*F*(*z*_*k*−1_) and *z*_∗_ corresponds to **y**. The term −2*η* *Δ* ^*T*^(*z*_*k*_−*z*_∗_) can further be bounded by using the proximal full gradient update $\bar {\mathbf {z}_{k}} = \text {prox}_{\eta R}\left (\mathbf {z_{k-1}} - \eta \nabla F\left (\mathbf {z_{k-1}}\right)\right)$, If Cauchy-Schwarz inequality and the non-expansiveness of the proximal mapping (∥prox_*ηR*_(*x*)−prox_*ηR*_(*y*)∥≤∥*x*−*y*∥) are utilized, the following expression can be derived.

$$\begin{array}{*{20}l} -2\eta\Delta^{T} \left(\mathbf{z_{k}} - \mathbf{z_{*}}\right) &= -2\eta\Delta^{T} \left(\mathbf{z_{k}} - \mathbf{z_{*}} + \bar{\mathbf{z}_{k}} - \bar{\mathbf{z}_{k}} \right)\\ & \leq 2\eta \left\|\Delta\right\| \left\|\mathbf{z_{k}} - \bar{\mathbf{z}_{k}}\right\| \\ &\quad- 2\eta \Delta^{T} \left(\bar{\mathbf{z}_{k}} - \mathbf{z_{*}}\right) \end{array} $$

If we insert the definitions of *z*_*k*_=(*z*_*k*−1_−*η* *v*_*k*_) and $\bar {\mathbf {z}_{k}} = \left (\mathbf {z_{k-1}} - \eta \nabla F\left (\mathbf {z_{k-1}}\right)\right)$, we will have:

$$\begin{array}{*{20}l} -2\eta\Delta^{T} \left(\mathbf{z_{k}} - \mathbf{z_{*}}\right) \leq 2\eta^{2} \left\|\Delta \right\|^{2} -2\eta \Delta^{T} \left(\bar{\mathbf{z}_{k}} - \mathbf{z_{*}}\right). \end{array} $$

If we combine the result shown above with Eq. (9):

$$\begin{array}{*{20}l} \left\|\mathbf{z_{k}} - \mathbf{z_{*}}\right\|^{2} &\leq \left\|\mathbf{z_{k-1}} - \mathbf{z_{*}}\right\|^{2} - 2\eta \left(P\left(\mathbf{z_{k}}\right) - P\left(\mathbf{z_{*}}\right)\right) \\ &\quad+ 2\eta^{2} \left\|\Delta \right\|^{2} - 2\eta \Delta^{T} \left(\bar{\mathbf{z}_{k}} - \mathbf{z_{*}}\right). \end{array} $$

Now, expectations of both sides are taken with respect to *z*_*k*_.

$$\begin{array}{*{20}l} \mathbb{E} \left\{ \left\|\mathbf{z_{k}} - \mathbf{z_{*}} \right\| \right\} &\leq \left\|\mathbf{z_{k-1}} - \mathbf{z_{*}}\right\|^{2} +2\eta^{2} \mathbb{E} \left\{\left\|\Delta \right\|^{2}\right\} \\ & \quad- 2\eta \left(\mathbb{E} \left\{P\left(\mathbf{z_{k}}\right)\right\} - P\left(\mathbf{z_{*}}\right)\right) \\ &\quad- 2\eta \mathbb{E} \left\{\Delta^{T} \left(\bar{\mathbf{z}_{k}} - \mathbf{z_{*}}\right)\right\} \end{array} $$

Since $\bar {\mathbf {z}_{k}}$ and *z*_∗_ are independent from the variable *z*_*k*_; $\mathbb {E} \left \{\Delta ^{T} \left (\bar {\mathbf {z}_{k}} - \mathbf {z_{*}}\right)\right \} = \mathbb {E} \left \{\Delta ^{T}\right \}\left (\bar {\mathbf {z}_{k}} - \mathbf {z_{*}}\right) = 0$. Because $\mathbb {E} \left \{\Delta ^{T}\right \} = \mathbb {E} \left \{\mathbf {v_{k}} - \nabla F\left (\mathbf {z_{k-1}}\right)\right \} = \mathbb {E}\left \{\mathbf {v_{k}}\right \} - \nabla F\left (\mathbf {v_{k-1}}\right) = 0$. The variance of the gradient $\mathbb {E}\left \{\left \|\Delta \right \|^{2}\right \}$ is upper bounded in Prox-SVRG algorithm and we will use the result of Corollary 3 in [15] which is $\mathbb {E}\left \{\left \|\Delta \right \|^{2}\right \} \leq 4L_{Q} \left [P\left (\mathbf {z_{k-1}}\right) - P\left (\mathbf {z_{*}}\right) + P\left (\tilde {\mathbf {z}}\right)-P\left (\mathbf {z_{*}}\right)\right ]$, where *L*_*Q*_= max*iL*_*i*_, $\tilde {\mathbf {z}}_{s} = \frac {1}{m}\sum _{k=1}^{m} \mathbf {z_{k}}$, and $\tilde {\mathbf {z}} = \tilde {\mathbf {z}}_{s-1} = \mathbf {z_{0}}$ for a fixed epoch. After incorporating the bound of the variance of the gradient into the analysis, the following expression is obtained.

$$\begin{array}{*{20}l} \mathbb{E}\left\{\left\|\mathbf{z_{k}}- \mathbf{z_{*}}\right\|^{2} \right\} &\leq \left\|\mathbf{z_{k-1}}- \mathbf{z_{*}}\right\|^{2} \\ &\quad- 2\eta \left(\mathbb{E}\left\{P\left(\mathbf{z_{k}}\right)\right\} -P\left(\mathbf{z_{*}}\right)\right) \\ &\quad+ 8\eta^{2}L_{Q} \left[P\left(\mathbf{z_{k-1}}\right) - P\left(\mathbf{z_{*}}\right)\right] \\ &\quad+ 8\eta^{2}L_{Q} \left[P\left(\tilde{\mathbf{z}}\right)-P\left(\mathbf{z_{*}}\right)\right] \end{array} $$

Now, if we apply the inequality above repeatedly for *k*=1,…,*m* and the expectation with respect to previous random variables *z*_1_,…,*z*_*m*_ are taken, then we can obtain the following inequality.

$$\begin{array}{*{20}l} &\mathbb{E}\left\{\left\|\mathbf{z_{m}}- \mathbf{z_{*}}\right\|^{2} \right\} + 2\eta\left[\mathbb{E}\left\{P\left(\mathbf{z_{m}}\right)\right\} - P\left(\mathbf{z_{*}}\right)\right]\\ &\quad+ 2\eta \left(1-4\eta L_{Q}\right)\sum_{k=1}^{m-1}\left[\mathbb{E}\left\{P\left(\mathbf{z_{k}}\right)\right\} - P\left(\mathbf{z_{*}}\right)\right]\\ &\leq \left\|\mathbf{z_{0}}- \mathbf{z_{*}}\right\|^{2}\\ &\quad+ 8 \eta^{2}L_{Q} \left[P\left(\mathbf{z_{0}}\right) - P\left(\mathbf{z_{*}}\right) +m\left(P\left(\tilde{\mathbf{z}}\right) - P\left(\mathbf{z_{*}}\right)\right)\right] \end{array} $$

Since 2*η*(1−4*η* *L*_*Q*_)<2*η*, $\mathbf {z_{0}} = \tilde {\mathbf {z}}$ and *P* is convex, therefore, $P\left (\tilde {\mathbf {z}}_{s}\right) \leq \frac {1}{m}\sum _{k=1}^{m}P\left (\mathbf {z_{k}}\right)$, and we can write the following inequality.

$$\begin{array}{*{20}l} & 2\eta \left(1-4\eta L_{Q}\right) m \left[\mathbb{E}\left\{P\left(\tilde{\mathbf{z}}_{s}\right)\right\} - P\left(\mathbf{z_{*}}\right)\right] \\ & \leq \left\|\tilde{\mathbf{z}}_{s-1} - \mathbf{z_{*}}\right\|^{2} \\ &\quad+ 8 \eta^{2} L_{Q}\left(m+1\right)\left(P\left(\tilde{\mathbf{z}}_{s-1}\right) - P\left(\mathbf{z_{*}}\right) \right) \end{array} $$

By using Lemma 1 which is a weaker condition then using the strong convexity and by applying the above inequality recursively, we derive the convergence rate as follows:

$$\begin{array}{*{20}l} & \left[\mathbb{E}\left\{P\left(\tilde{\mathbf{z}}_{s}\right) - P\left(\mathbf{z_{*}}\right)\right\}\right]\\ & \leq \left(\frac{\left(\frac{2}{\ell} + 8\eta^{2}L_{Q}\left(m+1\right)\right)}{2\eta \left(1-4\eta L_{Q}\right)m}\right)^{s}\left[P\left(\tilde{\mathbf{z}}_{0}\right)-P\left(\mathbf{z_{*}}\right)\right]. \end{array} $$

□

### **Lemma 1**

Consider the problem of minimizing the sum of two convex functions:

$$\min_{\mathbf{z} \in \mathbb{R}^{d}} \left\{P\left(\mathbf{z}\right) = F\left(\mathbf{z}\right) + R\left(\mathbf{z}\right)\right\}. $$

A standard method for solving the above problem is the proximal gradient method. Given an initial point *z*_0_, using the proximal mapping, which is shown below, iteratively generates a sequence that will converge to the optimal solution.

$$\text{prox}_{R}\left(\mathbf{y}\right) = \arg\min_{\mathbf{z} \in \mathbb{R}^{d}} \left\{\frac{1}{2} \left\|\mathbf{z}-\mathbf{y}\right\|^{2} + R(\mathbf{z}) \right\} $$

Since *R*(**x**) is a convex function, the optimal solution of above problem is also an optimal solution of the following problem using a tuning parameter *μ* *[* 30 *]* *[Theorem 1]*.

$$\min \frac{1}{2}\left\|\mathbf{z} - \mathbf{y}\right\|_{2}^{2} \ s.t. \ R\left(\mathbf{z}\right) \leq \mu $$

By utilizing the optimal strong convexity condition which is a weaker condition than strong convexity *[* 31 *]* for a convex function *R*, we have the following inequality for all **z**∈*Ω*:

$$P\left(\mathbf{z}\right) - P\left(\text{prox}_{E}\left(\mathbf{z}\right)\right) \geq \frac{\ell}{2}\left\|\mathbf{z} - \text{prox}_{E}\left(\mathbf{z}\right)\right\|^{2} $$

where the prox_*E*_ is the Euclidean projection on to set *E* and *ℓ* is a positive parameter.

We have thus removed the strong convexity condition so that we are able to apply the algorithm in [15] to more generic convex objectives.

## Results

In this section, we present the results of two types of experiments. First, the proposed algorithm was tested on synthetic datasets to investigate the convergence of the variance reduced proximal stochastic gradient compared to traditional proximal stochastic gradient descent. In addition, running times of the proposed stochastic Cvx-SPCA and other sparse PCA methods were compared to emphasize the advantage of using a stochastic approach, when there are large number of samples. In our experiments, step size *η* was chosen by the following heuristic according to 0<*η*<1/(4*L*_*Q*_) and *L*_*Q*_ was taken as the largest eigenvalue of the covariance matrix. Iteration number *m* was chosen as *Θ*(*L*_*Q*_/(*λ*−*λ*_1_(**S**))) which is suggested in [15]. Secondly, we presented our experiments on an electronic medical records data.

### Synthetic dataset

**Fig. 1:**
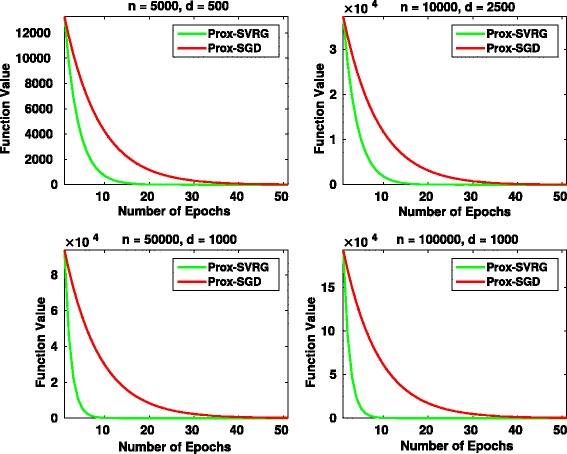
Convergence for synthetic data. Convergence of the proposed stochastic Cvx-SPCA with (Prox-SVRG) and without variance reduction (prox-SGD). Proximal stochastic gradient with variance reduction has a faster convergence rate, since the variance caused by random sampling is bounded in Prox-SVRG

In this section, we present some results of the proposed stochastic Cvx-SPCA algorithm on synthetic datasets. Synthetic datasets used in this section were all randomly generated by normally distributed random numbers with $\mathcal {N}\left (0,1\right)$. For this purpose, synthetic data with varying sample sizes were prepared by random sampling. First of all, we would like to compare the convergence of proximal stochastic gradient with variance reduction and traditional proximal stochastic gradient for our algorithm. In Fig. 1, objective versus number of epochs are plotted for using traditional proximal stochastic gradient (prox-SGD) and proximal stochastic variance reduced gradient (Prox-SVRG) methods.

**Fig. 2:**
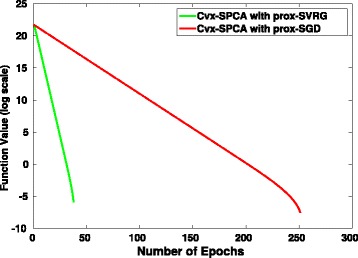
Convergence of sparse pattern in the log scale. Cvx-SPCA with Prox-SGD takes 275 iterations, whereas Cvx-SPCA with Prox-SVRG takes 45 iterations to converge a similar sparsity pattern

In Fig. 1, convergence is observed when the maximum number of epochs is fixed to 50. We also would like to investigate how many epochs are necessary for both algorithms to converge. Therefore, we made another experiment to see how fast Cvx-SPCA with Prox-SVRG converges to a similar sparsity as Cvx-SPCA with prox-SGD. We generated another synthetic dataset with 100,000 instances and 10,000 dimensions. The result of the experiment is shown in Fig. 2. Cvx-SPCA with traditional SGD took 3646.94 s and Cvx-SPCA with SVRG took 644.60 s to converge to similar sparsity patterns.

Secondly, running times of other sparse PCA methods and the proposed method were compared in Table 1. In experiments, feature dimension was chosen as 1000. Algorithms ran until they reached similar sparsity patterns. The proposed Cvx-SPCA algorithm is more scalable, since only one gradient is computed at a time and there are no eigenvalue decomposition or SVD steps during iterations. For instance, [9] requires singular value decomposition at each iteration, which is a bottleneck in terms of running time, [12] is an inverse power method based approach, and [11] uses semi-definite programming. Therefore, scalability with respect to sample size and dimension is an issue for the aforementioned methods.

**Table 1:** Running times (in seconds) of different SPCA algorithms

Sample size	Cvx-SPCA	[9]	[12]	[11]
*n*=50 k	20.9	207.1	48.7	3002
*n*=100k	26.2	466.9	78.3	3237.4
*n*=500 k	35.6	2737.06	2661.7	5276.93
*n*=1m	35.8	3408.59	3568	5274.26

Since proposed Cvx-SPCA does not depend on eigenvalue decomposition or semi-definite programming, it is more scalable in terms of the sample size. It also requires less iterations to reach a desired sparsity

**Fig. 3:**
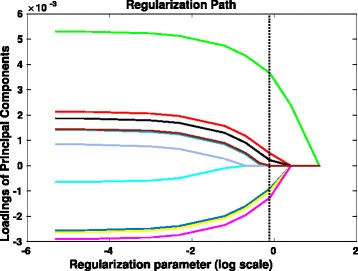
Regularization path for Cvx-SPCA. We checked whether the known principal component can be recovered through the path to be able confirm that this is a valid regularization path. When regularization term was around −0.11 (*dashed line*) in logarithmic scale, we could exactly recover the non-zero loading values of the known principal component which was used to generate the data

We also investigate the regularization path for the proposed algorithm. Regularization path illustrates how the solution changes for different values of regularization parameters *γ* which specify the level of sparsity. In order to have a suitable level of sparsity, *γ* should be tuned. One common way of finding an appropriate *γ* is the regularization path. We first generated a random sample with ten features and applied the proposed Cvx-SPCA algorithm to obtain the principal component. Then, the covariance matrix was reconstructed by using the first principal component corresponding to the largest eigenvalue with a little random noise. Loading values of principal components were computed with varying regularization parameters *γ* by using the reconstructed covariance matrix. We started with small *γ* values, and the loading vector learned from the previous step is used as the initialization for each new Cvx-SPCA step. The result is given in Fig. 3.

### Large-scale healthcare dataset

**Fig. 4:**
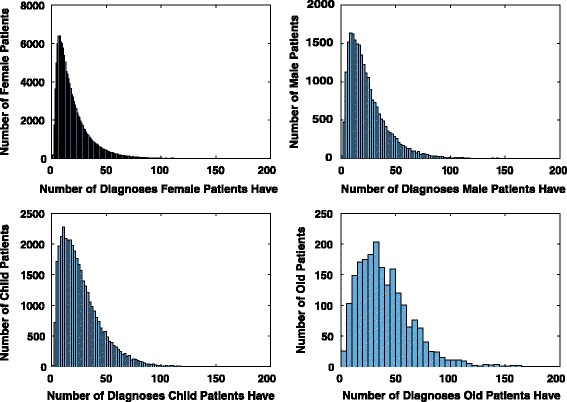
Patient distribution of demographic groups. We used only diagnoses/diseases which have explicit information about demographic of the patient while sub-sampling the patients. We can observe that each group of patient has a similar trend. Most of the patients have 1–50 diagnoses entered into the record

**Fig. 5:**
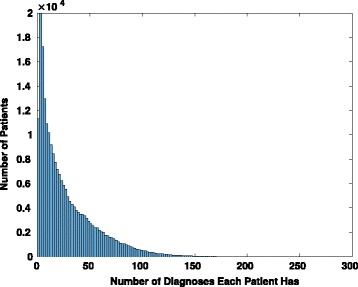
Patient distribution. We observe that the majority of the patients just have very few records

We applied our Cvx-SPCA algorithm to analyze disease patterns in a general patient population. The dataset we used is a real world electronic medical record (EMR) warehouse including the records of 223,076 patients over 4 years. We used the diagnosis information (in terms of ICD9 codes [32]) in our investigation, which resulted in 11,982 features in total. In this dataset, we do not have demographic information of patients explicitly. However, we investigated patient groups with different gender and age by looking at the descriptions of the ICD9 codes. We draw histograms of the number of patients with respect to the number of diagnoses each patient has in different demographic groups and in the general population as in Figs. 4 and 5, from which we can observe that the majority of the patients just have very few records. In our experiments, we eliminated the patients who have less than five records, and this resulted in 177,856 patients. As it was mentioned earlier, some of the diseases are specifically related to gender and age that let us have an idea about the demographic information of the dataset. For instance, complications of pregnancy, female genital disorders, and abortion are some of the diagnoses which are explicitly about women. Similarly, maternal complications affecting newborn and diseases such as chickenpox and measles are related to children. There are also ICD9 codes which have terms indicating the age. For instance, some of the diagnoses have the term “senile” which points out patients at least above 60 years old. Thus, we sampled female, male, old, and child patients by taking the definitions of the ICD9 codes into account. The age range of child patients can be given as from babyhood to adolescence and age of old patients can be thought as above 60 years old. In Table 2, number of patients and number of features related to female, male, people above 60 years old and children groups are given. We should note that there may be female, male, old, and child patients who we did not include into these demographic groups. For example, there should be female/male patients with diagnoses which are not gender- or age-specific. It is not always possible to guess the gender or age from diagnosis such as hypertension or infectious diseases which can be encountered in both genders. Therefore, we are reporting the demographic groups whose ICD9 codes have clear terms indicating the demographic information.

**Table 2:** We sample patients who have female, male, child, and old people related features. These samples may overlap with each other. For instance, a patient may have dementia and a prostate problem together. We did not include other problems such as hypertension or kidney problems which can be encountered in every age and both genders into these groups of patients

Demographic	Number of features	Number of patients
Female	1268	130,035
Male	106	24,184
Old	66	2060
Child	596	38,434

As can be seen from Table 2, the number of female-specific diseases and the number of female patients are more than other groups in the EMR dataset we used in this paper. Number of old patients is given less than other groups in the table. However, it may not mean that there are less number of old people in the whole patient population. We could not exactly extract age information of every diagnoses/diseases. For instance, hypertension or Alzheimer’s were diseases commonly encountered among the people above a certain age in the past. However, these problems can be occurred in younger ages recently. For this reason, we used only diagnoses/diseases which have explicit information about demographic of the patient, while sub-sampling the patients. Distributions of different patient groups in Table 2 are given in Fig. 4.

In our experiments, we further aggregated all diagnoses belong to the same ICD9 group together, so that each patient is represented by a 918 dimensional feature vector. The value on its *i*th dimension represents the frequency of the *i*th diagnosis code appearing in the EMR of the corresponding patient. Since every patient will have a limited number of diseases, patient vectors are very sparse.

**Fig. 6:**
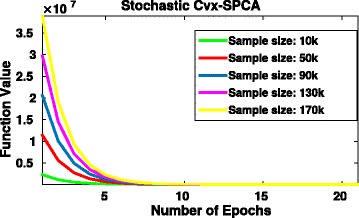
Convergence for 20 epochs of Cvx-SPCA for different number of patients

We would like to emphasize that existing sparse PCA algorithms cannot be used to analyze a dataset at this scale. We carried out both quantitative and qualitative evaluations on this dataset. We studied the convergence of the algorithm with varying number of patients, and we observe that the proposed Cvx-SPCA can still achieve a good convergence even when the sample size is very large, as shown in Fig. 6.

Next, we conducted an experiment to show how the proposed algorithm helps us to analyze the EMR data. We applied our algorithm to the whole data set and got the output features which correspond to the non-zero loading values of the leading principal component. These output features are inferred as key medical features. One of the results is summarized in Table 3. Diseases shown in this table are the features which have non-zero loadings whose absolute values are greater than a heuristic threshold. In our experiments, we observed that the most frequently encountered output features were infectious diseases, problems related to pregnancy and labor, injuries, and cancer types. This result tells us that the proposed algorithm can provide insight about the diagnoses encountered in the patient population.

**Table 3:** EMR data features which contributes the output dimensions after Cvx-SPCA algorithm was applied to the whole patient population. Most frequently observed problems are infections, injuries, pregnancy, and delivery related problems and cancer types

ICD9 code	Description
7	Balantidiasis/infectious
72	Mumps orchitis/infectious
115	Infection by histoplasma capsulatum
266	Ariboflavinosis/metabolic disorder
507	Pneumonitis/bacterial
695	Toxic erythema/dermatological
697	Lichen planus/dermatological
761	Incompetent cervix affecting fetus or newborn
795	Abnormal glandular papanicolaou smear of cervix
924	Contusion of thigh/injury

We further examined the data set and divided the features into groups in terms of gender and age. We sampled the patients who have gender- and age-related problems separately and applied our algorithm to those samples to analyze the output dimensions. Examples from each group are shown in Tables 4, 5, 6, and 7. We can see plausible results for the output features of each group in the tables. For example, diagnoses such as female genital disorders, perinatal problems, and anemia, which are more common among women, appeared in Table 4 where the algorithm was applied to the subset of patients who have female-related problems. Similarly, we can see from Table 5 that a subset of male patients generates prostate cancer along with other diagnoses which can be frequently seen in the general patient population as well. Cancer is a commonly encountered problem in nearly every age. We can come across cancer in the results of children and old patients as well. Another observation is that tuberculosis and bacterial infections are quite common among children.

**Table 4:** Output EMR data features which contributes the output dimensions after applying the proposed algorithm to the subset of patients who have female-related problems. We could observe female-specific problems and other common diseases such as heart problems and anemia

ICD9 code	Description
281	Pernicious anemia
392	Valvular and rheumatic heart disease
614	Female genital disorders
778	Serious perinatal problem affecting newborn
905	Major head injury

**Table 5:** Output EMR data features which contributes the output dimensions after applying the proposed algorithm to the subset of patients who have male related problems. We could observe a prostate problem which is directly related male patients. In addition, we can also see other common problems such as injuries

ICD9 code	Description
185	Malignant neoplasm of prostate
298	Depressive type psychosis
719	Effusion of joint
800	Closed fracture of vault of skull
811	Closed fracture of scapula
860	Traumatic pneumothorax

**Table 6:** Output EMR data features which contributes the output dimensions after applying the proposed algorithm to the subset of patients who have old age-related problems. Cancer is a commonly encountered problem in nearly every ages. In addition to this, we could observe disorders of nervous system and visual problems in the results

ICD9 code	Description
153	Malignant neoplasm of colon
173	Other malignant neoplasm of skin
337	Disorders of the autonomic nervous system
368	Visual disturbance

**Table 7:** Output EMR data features which contributes the output dimensions after applying the proposed algorithm to the subset of patients who have child related problems. According to our observation, tuberculosis and bacterial infections are quite common among children. Unfortunately, leukemia is also a cancer type that is seen even in small kids

ICD9 code	Description
8	Intestinal infection due to other organisms
11	Pulmonary tuberculosis
78	Other diseases due to viruses and Chlamydiae
10	Primary tuberculous infection
204	Lymphoid leukemia

## Discussion

Throughout the paper, advantage of using a convex optimization approach for sparse PCA is emphasized. In this section, we would like to discuss about our conjuring of the convergence of non-convex stochastic sparse PCA by using the same framework. One surprising finding we have is if we use this non-convex PCA to construct a non-convex sparse PCA (by adding *ℓ*_1_-norm), we still benefit from a much faster convergence rate using the stochastic scheme studied in this paper. A similar result is also presented in [7], where the authors propose a stochastic PCA approach with an exponential convergence rate by using variance reduced stochastic gradient presented in [14]. These results lead us to ask the following question: *Can we generalize the convergence analysis of proximal variance reduced stochastic gradient method further for non-convex settings?* We will investigate this problem in the future work.

## Conclusions

In this paper, a convex stochastic sparse PCA method is proposed. Since the problem of finding the leading eigenvector is formed as convex optimization, a well-defined convergence rate can be applied to the proposed algorithm. A proximal stochastic gradient method with variance reduction is preferred to avoid low convergence rates of traditional stochastic methods. Although strong convexity is usually required in literature, we simplify the convergence analysis of the existing Prox-SVRG algorithm by using weaker conditions. According to the experiments on several synthetic data, the proposed algorithm is shown to be more scalable due to stochastic approach. In addition, an application of sparse PCA is presented to show how sparse PCA can help to interpret electronic medical records. In future work, we would like to investigate whether sparse PCA can be used to cluster patients with respect to their medical records. For instance, we propose to apply the proposed algorithm to analyze medical records and derive clinically meaningful and structural phenotypes, which can further be helpful for patient risk stratification and clustering.
